# A Novel TNM Classification for Colorectal Cancers based on the Metro-ticket Paradigm

**DOI:** 10.7150/jca.55097

**Published:** 2021-04-05

**Authors:** Jun-Peng Pei, Chun-Dong Zhang, Xiang Fu, Yong Ba, Shuai Yue, Zhe-Ming Zhao, Dong-Qiu Dai

**Affiliations:** 1 Department of Gastrointestinal Surgery, The Fourth Affiliated Hospital of China Medical University, Shenyang 110032, China.; 2 Department of Gastrointestinal Surgery, Graduate School of Medicine, The University of Tokyo, Tokyo 113-0033, Japan.; 3 Cancer Center, The Fourth Affiliated Hospital of China Medical University, Shenyang 110032, China.

**Keywords:** colorectal cancer, TNM classification, metro-ticket, novel TNM classification

## Abstract

**Background:** Several revisions of the TNM classifications for colorectal cancer (CRC) have acknowledged that the oncological outcomes of stage IIB/IIC CRC are worse than those of stage IIIA. We aimed to develop a novel TNM (nTNM) classification based on the metro-ticket paradigm.

**Methods:** We identified eligible CRC patients from the Surveillance, Epidemiology, and End Results database. The nTNM was developed using distance from the origin on a Cartesian plane incorporating the pN (x-axis) and pT (y-axis) stages, and was compared with the AJCC TNM classification. The areas under the curves (AUCs), calibration curves, and Akaike's information criterion (AIC) were used to evaluate the predictive performances of the two classifications. Clinical benefits were further estimated by decision curve analyses. The validation cohort was applied to validate these findings.

**Results:** A total of 58,192 CRC patients (40,736 training cohort, 17,456 validation cohort) were finally included. In the training cohort, 18,476 patients (45.4%) experienced upstaging and 15,907 patients (39.0%) experienced downstaging in the nTNM classification compared with the TNM classification. Taking the prognosis of stage I as the reference, survival decreased with increasing nTNM stage. The nTNM classification showed better discrimination (AUC, 0.678 vs. 0.667, *P*<0.001), model-fitting (AIC, 236,525 vs. 237,741), and clinical benefits than the TNM classification. Similar results were found in the validation cohort.

**Conclusions:** The nTNM classification for CRC has better predictive performances and superior accuracy for predicting prognosis compared with the TNM classification. The nTNM classification should therefore be considered in future revisions of the TNM classification.

## Introduction

Colorectal cancer (CRC) is the third most commonly diagnosed cancer among both males and females in the United States 1. The American Joint Committee on Cancer (AJCC) tumor-node-metastasis (TNM) staging system is the most widely used prognostic standard for CRC 2, and has been revised several times over the past few decades to improve its prognostic performance and treatment suggestions for patients with CRC. In general, the survival of patients with high-stage cancers should be lower than that of patients with low-stage cancers. However, CRC may be an exception, especially with respect to stage IIB/C (T4N0) and stage IIIA (T1-2N1, T1N2a) tumors, with an apparent survival paradox between these stages. Based on the AJCC 7^th^ and 8^th^ TNM staging systems, data from the Surveillance, Epidemiology, and End Results (SEER) program indicated that the survival of patients with stage IIB/IIC CRC was worse than that of those with stage IIIA 3,4. Similar results were also reported by the Japan Colon Cancer and Rectal Cancer Society and other research institutes 5-11.

Several recent studies have suggested a 'metro-ticket' paradigm to evaluate survival 12-16. This paradigm was originally proposed by Mazzaferro et al. for hepatocellular cancer (HCC) and later evolved into a novel predictive tool for HCC patients after liver transplantation 12. The metro-ticket paradigm was based on the concept that the longer the “trip” (i.e., increased T stage and N stage), the higher the price of the “ticket” (i.e. reduction in expected survival). Further research by Lu et al. showed that application of the metro-ticket system to the TNM staging system for gastric cancer could accurately stratify patients and improve its prognostic performance 15. However, no studies have yet applied the metro-ticket paradigm to the TNM staging system for CRC.

We aimed to exploit a novel TNM (nTNM) staging system for CRC based on the metro-ticket system, compare the prognostic performances of the nTNM and the AJCC 8^th^ TNM staging systems, and validate these findings in a validation cohort.

## Methods

### Patients

Screened colorectal cancer patients from the SEER program from 1973 to 2015. The inclusion criteria were as follows: patients with CRC; provided the complete clinical information; patients were between 18 and 72 years old; first and single cancer; no distant metastasis (M0); performed surgical treatment; no neoadjuvant radiotherapy and chemotherapy were performed; survive more than 1 month; and follow-up at least 5 years or until death. The exclusion criteria were as follows: no complete clinical information; aged< 18 or > 72 years; have other malignant tumors; distant metastasis (M1); no surgical treatment; performed neoadjuvant radiotherapy and chemotherapy; survival time after surgery were less than 1 month; and follow-up time were less than 5 years or lost to follow-up.

### Development of nTNM staging system

**Figure 1:**
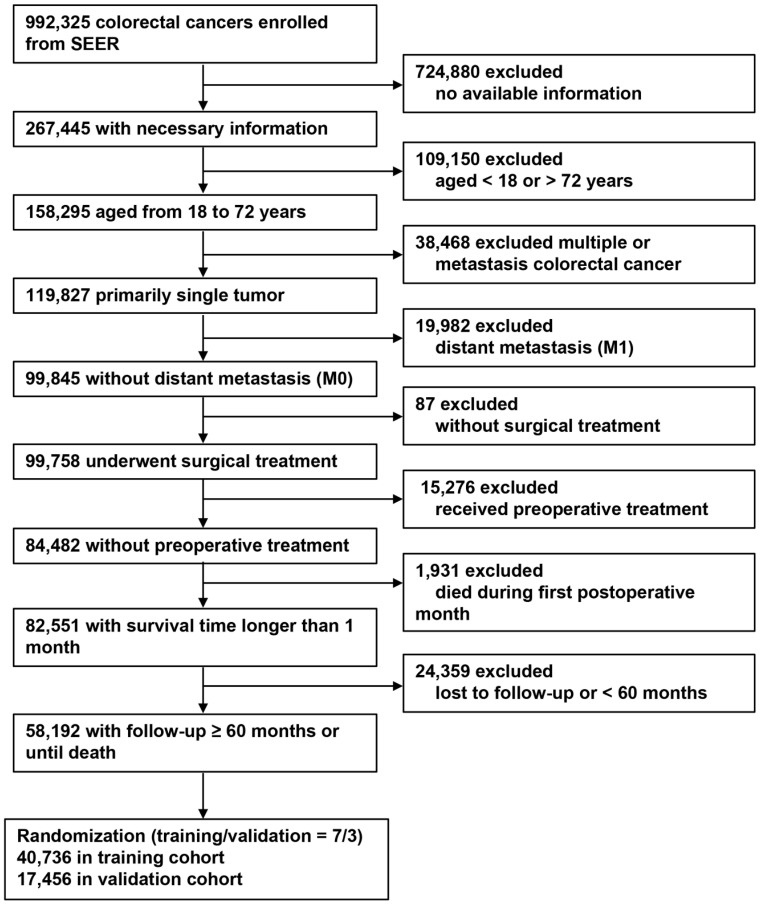
Flow chart for patient selection.

Based on the concept of the metro-ticket paradigm for gastric cancer, we proposed the current nTNM classification by combining the pN and pT stages, with preserved definitions of pN and pT stages. The nTNM was defined as the distance from the origin on a Cartesian plane that incorporated two variables: the pN stage (x-axis) and pT stage (y-axis); Pythagoras theorem was then used to calculate the distance of any given point from the origin of the plane (0, 0), where [(nTNM)^2^ = (pN)^2^ + (pT)^2^] (Figure 1A, 1B) 12-16. The nTNM classification was similar to the AJCC classification and was divided into seven subgroups: I, IIA, IIB, IIC, IIIA, IIIB, and IIIC.

### Statistical analysis

Categorical and continuous variables were described as frequency (%) and median (interquartile range [IQR]), respectively. The characteristics of the baseline clinical variables between the training and validation cohorts were compared with Student's *t*-test or χ^2^ test. Five-year overall survival (OS) and estimations of hazard ratios (HRs) were calculated and presented with 95% confidence intervals (CI). The prognostic performance of the nTNM was compared with that of the AJCC 8^th^ TNM classification. The Kaplan-Meier method with log-rank tests was applied. The model discrimination abilities of the nTNM and AJCC TNM staging systems were assessed by areas under the curves (AUCs), and the AUCs were compared by Hanley & McNeil tests. Akaike's information criterion (AIC) was applied to evaluate the model-fitting 17. Higher AUCs indicated better model discrimination, and lower values of AIC indicated superior model-fitting. Calibration curves were used to determine the agreement between the predicted and observed survival probabilities; in a perfect model, the predictions should fall on the diagonal 45° line of the calibration curve 18. Clinical benefit was further evaluated by decision curve analysis (DCA) 19.

All data were analyzed using MedCalc (Version 15.2, Ostend, Belgium), SPSS 22.0 (SPSS Inc., Chicago, IL, USA), and R version 3.6.1. All tests were two-sided and *P* values < 0.05 were considered statistically significant.

## Results

### Patient characteristics

Based on the inclusion and exclusion criteria, 58,192 patients with non-metastatic CRC were finally included and divided into a training (n = 40,736) and a validation cohort (n = 17,456) using a random sampling method (sampling ratio 7:3). The screening process is shown in Figure 1. In the training cohort, the median number of retrieved lymph nodes was 16.0 (IQR, 30.0-65.0). A total of 16,623 CRC patients had lymph node metastasis, with a median number of positive lymph nodes of 3.0 (IQR, 1.0-5.0). Tumor sizes were recorded for 37,033 patients, with a median of 4.0 cm (IQR, 3.0-6.0 cm). A total of 31,947 (78.4%) patients had colon cancer and 8,789 (21.6%) had rectal cancer. The clinical and pathological characteristics of the validation cohort were similar to those of the training cohort. Table 1 shows the detailed clinical and pathological characteristics of the training and validation cohorts.

### The nTNM staging system

**Figure 2:**
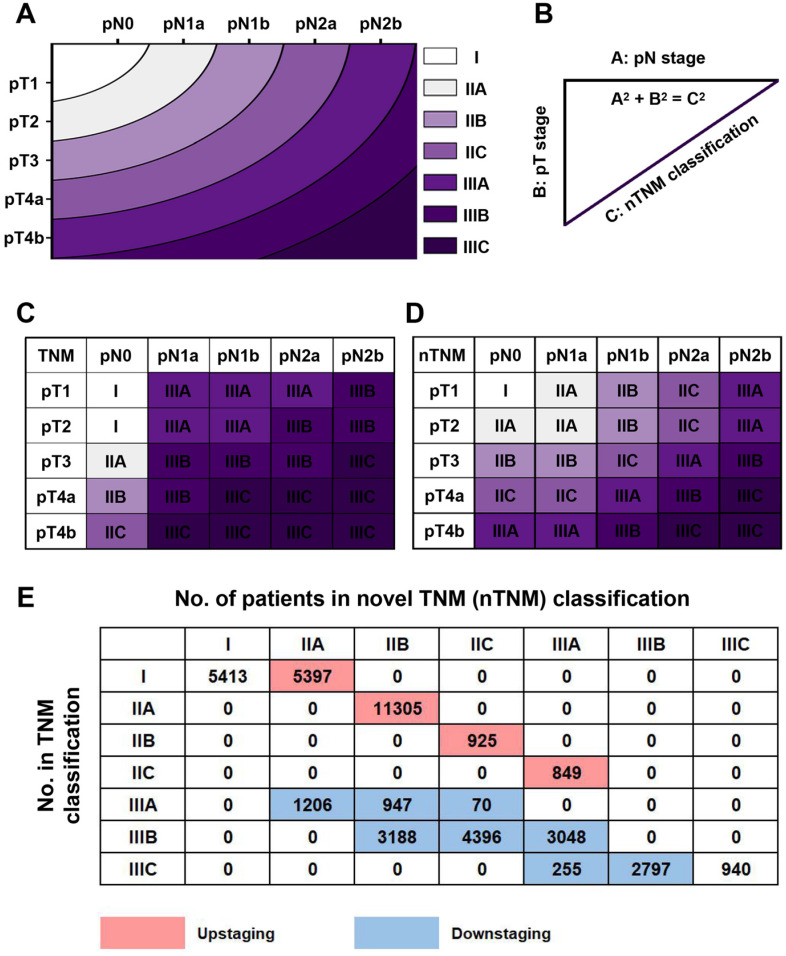
A: the novel TNM (nTNM) classification was defined as the distance from the origin on a Cartesian plane that incorporated two variables: the pN stage (x-axis) and pT stage (y-axis); B: the Pythagoras theorem was used to calculate the distance of any given point from the origin of the plane (0, 0), where [(nTNM)2 = (pN)^2^ + (pT)^2^]; C: AJCC 8^th^ TNM classification; D: nTNM classification; E: the staging migration between the nTNM and AJCC 8^th^ TNM staging systems in the training cohort.

The principle of the nTNM staging system involves using the distance from the origin on the Cartesian plane (Figure 2A, 2B). An intuitional comparison of the AJCC 8^th^ TNM and nTNM staging systems is shown in Figure 2C and 2D. The nTNM showed significant changes compared with the AJCC 8^th^ TNM staging system (Figure 2E). In the training cohort, 18,476 patients (45.4%) experienced upstaging in the TNM classification, including 5,397 patients (13.2%) in stage I, 11,305 patients (27.8%) in stage IIA, 925 patients (2.3%) in stage IIB, and 849 patients (2.1%) in stage IIC; while 15,907 patients (39.0%) experienced downstaging, including 2,220 patients (5.5%) in stage IIIA, 10,632 patients (26.1%) in stage IIIB, and 3,025 patients (7.5%) in stage IIIC (Figure 2E).

### OS based on AJCC TNM and nTNM staging systems

**Figure 3:**
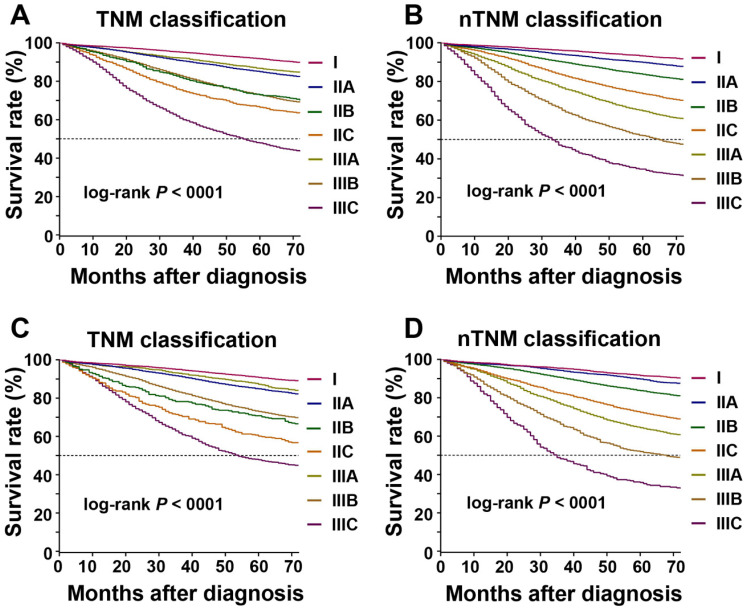
Kaplan-Meier survival curve for overall survival according to the AJCC 8^th^ TNM and novel TNM (nTNM) staging systems. A: the AJCC 8^th^ TNM staging system in the training cohort; B: the nTNM staging system in the training cohort; C: the AJCC 8^th^ TNM staging system in the validation cohort; D: the nTNM staging system in the validation cohort.

According to AJCC 8^th^ TNM staging system, the 5-year OS rates for each stage in the training cohort were: I, 91.7%; IIA, 85.0%; IIB, 73.0%; IIC, 66.5%; IIIA, 86.7%; IIIB, 72.9%; and IIIC, 47.9% (log-rank test, IIB vs IIIB: *P* = 0.875, *P* < 0.005 for the other stages; Figure 3A). Kaplan-Meier analysis revealed a higher 5-year OS in patients with stage IIIA than in those with stages IIB and IIC (log-rank test, both *P* < 0.001), higher 5-year OS in stage IIIB than stage IIC (log-rank test, *P* < 0.001), and similar 5-year OS in stage IIB and stage IIIB (log-rank test, *P* = 0.875). For the nTNM classification, the 5-year OS rates for each stage were: I, 93.2%; IIA, 89.9%; IIB, 83.7%; IIC, 73.7%; IIIA, 64.8%; IIIB, 52.0%; and IIIC, 34.7% (log-rank test, *P* < 0.001 for all stages; Figure 3B). The 5-year OS rates increased sequentially from stages I to IIIC.

In the training cohort, the HRs of OS for each stage of the AJCC 8^th^ TNM staging systems were as follows: IIA, 1.665; IIB, 2.818; IIC, 3.540; IIIA, 1.364; IIIB, 2.839; and IIIC, 6.328 (stage I as the reference) (Table 2 and Figure 3). The HR for patients with stage IIIA was significantly lower than those for stages IIA, IIB, and IIC (all *P* < 0.001), the HR for stage IIIB was higher than for stage IIC (*P* < 0.001), and the HRs for stage IIB stage IIIB were similar (log-rank test, *P* = 0.875). HRs for each stage of the nTNM classification: IIA, 1.441; IIB, 2.151; IIC, 3.326; IIIA, 4.609; IIIB, 6.855; and IIIC, 11.385 (stage I as the reference). The HRs increased sequentially from stages I to IIIC. Similar results were also observed in patient groups with insufficient (< 12) and sufficient (≥ 12) numbers of retrieved lymph nodes (Table S1 and Figure S1). Similar results were confirmed in the validation cohort (Table 2, Table S1, Figure 3C, 3D, and Figure S2).

### Comparison of prognostic performances between AJCC 8^th^ TNM and nTNM staging systems

**Figure 4:**
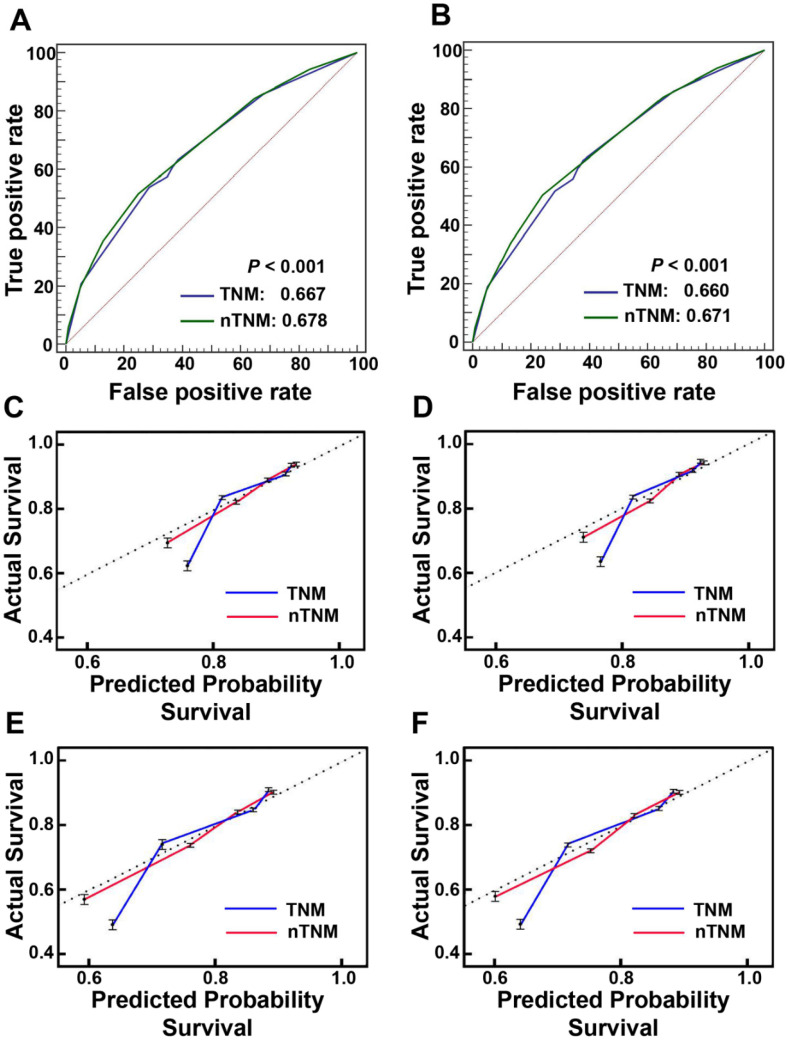
The areas under the curves (AUCs) and calibration curves for predicting patient survival. A: AUCs of the AJCC 8^th^ TNM staging system and novel TNM (nTNM) staging system in the training cohort; B: AUCs of the AJCC 8^th^ TNM staging system and nTNM staging system in the validation cohort; C, At three-year overall survival (OS) in the training cohort; D, At three-year OS in the validation cohort; E, At five-year OS in the training cohort; F, At five-year OS in the validation cohort.

The prognostic discrimination of the nTNM in the training cohort (AUC 0.678, 95% confidence interval (CI), 0.673-0.682) was superior to that of the AJCC 8^th^ TNM classification (AUC 0.667, 95% CI, 0.662-0.672) (Hanley & McNeil test, *P* < 0.001). The nTNM also had better model-fitting than the AJCC 8^th^ TNM classification (AIC, 236,525 vs. 237,741) (Table 3, Figure 4A). The calibration curves for 3-, and 5-year OS in the nTNM staging system also showed better agreement than the AJCC 8th TNM staging system (Figure 4C, 4D). Similar results were confirmed in the validation cohort (Table 3, Figure 4B and Figure 4E, 4F).

### Clinical usefulness

**Figure 5:**
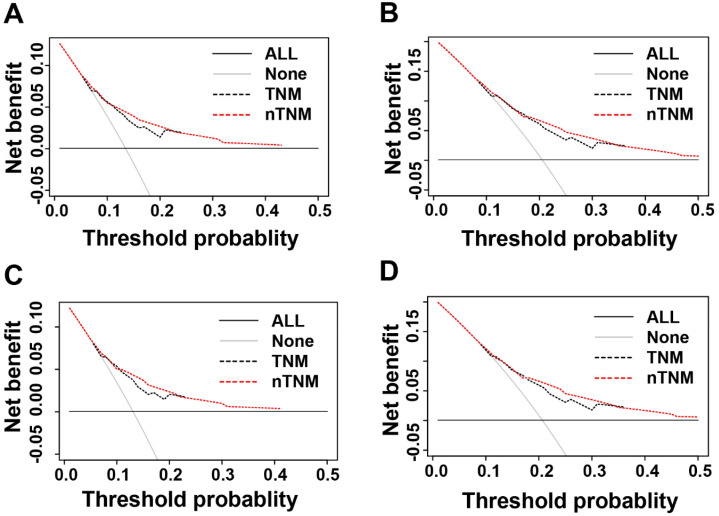
Decision curve analysis (DCA) of three- and five-year overall survival (OS) of novel TNM (nTNM) staging system and AJCC 8^th^ TNM staging system. A: the three-year OS in the training cohort; B: the five-year OS in the training cohort; C: the three-year OS in the validation cohort; D: the five-year OS in the validation cohort.

We evaluated the clinical usefulness of the AJCC 8^th^ TNM and nTNM staging systems in the training and validation cohorts by DCAs. In the training and validation cohorts, the net benefit of the nTNM staging system was higher than that of the AJCC 8th TNM staging system between threshold probabilities of around 10%-20% in predicting 3-year OS, and between threshold probabilities of around 20%-35% in predicting 5-year OS (Figure 5).

## Discussion

Accurate cancer staging helps doctors to predict survival and provide more effective therapeutic recommendations. According to the AJCC TNM classifications, the survival of patients with low-stage cancers is generally higher than that for high-stage cancers among patients with most solid malignant tumors 20. However, CRC is one of the few exceptions, and many studies have shown an obvious survival paradox between patients with stage IIB/IIC and stage IIIA CRC 6,8-11. The current study confirmed that the survival of patients with stage IIIA CRC was lower than that of patients with stage IIB/IIC CRC.

Researchers have proposed numerous explanations for this paradox, including possible staging migration as a result of retrieval of insufficient lymph nodes 21. This means that the survival of patients with stage IIB/C disease who undergo sufficient lymphadenectomy would be better than that of patients with stage IIIA CRC. However, previous studies showed that the survival paradox persisted, even if sufficient lymph nodes were retrieved 6,9. Some researchers also attributed the survival paradox to preferential receipt of adjuvant radiotherapy and chemotherapy in stage IIIA disease or a lack of systemic treatment in stage IIB/C CRC 21,22. The role of postoperative adjuvant chemotherapy in patients with stage II CRC remains controversial. The American Society of Clinical Oncology recommends postoperative adjuvant chemotherapy for patients with stage T4 CRC 23, while several studies have shown that patients with stage IIB/C CRC who receive adjuvant chemotherapy still have significantly lower survival than stage IIIA patients 6,9,11. An inadequate use of postoperative chemotherapy therefore cannot explain the poor prognosis of patients with stage IIB/C CRC. Another possibility is that the poor prognosis of stage IIB/C CRC may be related to the presence of residual tumor after resection 6,7,24. Negative margins can be achieved more easily for T1-T2 lesions (i.e., stage IIIA), while extensive en-bloc resection should be performed for T4 lesions (i.e., stage IIB/C), but such surgery is more complicated. Resection of stage IIB/C tumors is thus more likely to result in positive margins than resection of stage IIIA tumors. Nonetheless, among patients with positive margins, the survival of patients with stage IIB/C CRC was still lower than that of stage IIIA, and the survival of patients with stage IIB/C and negative margins was similar to that of patients with stage IIIA and positive margins 6,7.

This paradox is widely thought to be caused by the inherent biological invasiveness of stage IIB/C CRC, with various lines of evidence to support this view. Perineural invasion, a high-frequency of microsatellite instability and the tumor's inflammatory reaction can all increase the biological aggressiveness of the tumor. Previous studies showed that stage IIB/C CRC had a higher proportion of perineural invasion, a high frequency of microsatellite instability, and a more prominent protumor inflammatory reaction compared with stage IIIC CRC 9,11. Previous studies have shown characteristic differences between stages IIB/C and IIIA, with stage IIB/IIC cancers showing faster intestinal wall infiltration and slower lymph node metastasis, and stage IIIA cancers having slower intestinal wall infiltration and earlier lymphatic infiltration 9,11. This biological difference suggests that early stages do not translate into better oncological outcomes or less recurrence. Some researchers used gene expression profiling to stratify colon cancer according to the risk of recurrence, and indicated that stage IIA/C CRC had poor tumor prognosis and high recurrence rates 25,26.

Li et al. suggested that the survival paradox between stages IIB/IIC and IIIA CRC was derived from the inherent concept of the current TNM classification that lymph node metastases (N stage) affect the prognosis more significantly than local invasion (T stage) 27,28. In the past few decades, the AJCC system has undergone several revisions, and compared with the AJCC 6^th^ TNM classification for CRC, the 7^th^ TNM classification increased the weight of T stage. Patients with CRC that directly infiltrates or adheres to adjacent organs or structures have poor outcomes. The AJCC 7^th^ TNM classification therefore divides stage T4 into stage T4a (tumor penetrates the visceral peritoneal surface) and stage T4b (tumor directly invades or histologically adheres to adjacent organs or structures). T4bN0 lesions were reclassified from stage IIB to stage IIC and T1-2N2 lesions from IIIC to IIIB/IIIC 4,29. These modifications remained unchanged in the latest AJCC 8th TNM classification^4^, and mirrored the increased weighting of T stage in the TNM classification for CRC. However, Li et al. indicated that the current TNM classification still underestimates the weight of T stage and overestimates the weight of N stage 27,28. The above research showed that the survival paradox between IIB/IIC and IIIA CRC was essentially related to the inherent biological invasiveness of stage IIB/C, and the fact that the AJCC 8^th^ TNM classification underestimates the weight of T stage or overestimates the weight of N stage. We therefore proposed the nTNM classification based on the metro-ticket paradigm to resolve this survival paradox.

The metro-ticket paradigm was originally used to predict the long-term prognosis of patients undergoing liver transplantation for HCC^12^, and this prognostic model was shown to accurately stratify the survival of HCC patients. This theory was subsequently applied to patients with CRC liver metastases, thus establishing a novel tumor burden scoring system that could accurately predict patient prognosis after resection in patients with CRC liver metastases^14^. In another study, Lu et al. developed a new TNM gastric cancer classification based on the same theory and showed that this new system was superior to the AJCC 8^th^ TNM classification for predicting the long-term survival of patients with gastric cancer^15^. However, no study has yet developed a novel TNM classification for CRC based on the same theory. We therefore modeled pT and pN stages on a Cartesian plane and predicted the survival of CRC patients based on this model. Applying the principle of the metro-ticket paradigm, the weights of the pT and pN stages were considered equally.

Rice et al. summarized the characteristics of an excellent classification as: distinguishability between different stages, homogeneity in the same stage, and monotonicity of gradients 30. Regarding distinguishability, the nTNM showed significant differences in survival between each pair of stages (log-rank test, all *P* < 0.001), whereas the AJCC 8^th^ TNM classification showed no significant difference in survival between stages IIB and IIIB (log-rank test, *P* = 0.875). The nTNM classification also performed better than the AJCC 8^th^ TNM classification in terms of gradient monotonicity. The Kaplan-Meier survival curves indicated that the nTNM classification distinguished patients into different stages, with decreasing survival corresponding with increased stage; in contrast, the survival of stage IIB/C was higher than that of stage IIIA according to the AJCC 8^th^ TNM classification.

The nTNM also showed significantly better ability to predict prognosis and clinical utility than the AJCC 8^th^ TNM classification. In this study, the nTNM classification had a lower AIC value and a higher AUC value (Hanley & McNeil test, all *P* < 0.001) than the AJCC 8^th^ TNM classification. The calibration curve of survival probability also manifested better consistency between predicted and observed survival in the nTNM staging system. DCA analysis demonstrated that the nTNM staging system had better clinical benefits than the AJCC 8^th^ TNM staging system.

The large sample size of this study suggests that the results were reliable. However, the study also had some limitations. First, it was a retrospective study and may therefore have been affected by selection bias and unknown confounding factors. Further prospective studies and other large-scale multicenter data are therefore needed to verify our results. In addition, postoperative adjuvant therapy may affect the prognosis of patients with CRC; however, SEER data lacks information on adjuvant therapy, which may have had an impact on the prognostic ability of the TNM classification.

## Conclusions

The nTNM staging system has better prognostic performance than the AJCC TNM 8^th^ staging system for predicting the prognosis of patients with CRC. We therefore suggest that the nTNM staging system should be considered in future revisions of the AJCC TNM staging system. However, the findings of the current study require further external validation.

## Supplementary Material

Supplementary figures and tables.

## Figures and Tables

**Table 1:** Clinical and pathological characteristics of the training and validation cohorts

Variable	Univariate analysis (training cohort)			Univariate analysis (validation cohort)			*P* value*
	No. patients (%)	5-year OS	*P* value	No. patients (%)	5-year OS	*P* value	
**Age, year**			<0.001			<0.001	0.281
≤ 60	21747 (53.4)	82.6%		9234 (52.9)	83.1%		
> 60	18989 (46.6)	75.8%		8222 (47.1)	75.0%		
**Gender**			<0.001			<0.001	0.381
Female	18807 (46.2)	81.4%		8128 (46.6)	81.2%		
Male	21929 (53.8)	77.7%		9328 (53.4)	77.7%		
**Race**			<0.001			<0.001	0.632
White	31150 (76.5)	80.2%		13360 (76.5)	80.0%		
Black	5560 (13.6)	72.4%		2408 (13.8)	72.6%		
Other	4026 (9.9)	82.9%		1688 (9.7)	83.5%		
**Location**			0.895			0.808	0.228
Colon	31947 (78.4)	79.2%		13611 (78.0)	79.2%		
Rectum	8789 (21.6)	80.2%		3845 (22.0)	79.6%		
**Size, cm**			<0.001			<0.001	0.083
≤ 4	18747 (46.0)	82.4%		8064 (46.2)	82.1%		
> 4	18286 (44.9)	75.3%		7720 (44.2)	75.3%		
Unknown	3703 (9.1)	84.8%		1672 (9.6)	84.2%		
**Histological grade**			<0.001			<0.001	0.236
Grade I	3877 (9.5)	86.9%		1620 (9.3)	86.6%		
Grade II	29898 (73.4)	80.7%		12812 (73.4)	80.9%		
Grade III	6335 (15.6)	69.8%		2735 (15.7)	69.3%		
Grade IV	626 (1.5)	67.2%		289 (1.7)	64.0%		
**AJCC 8^th^ pT stage**			<0.001			<0.001	0.348
T1	6267 (15.4)	92.4%		2733 (15.7)	91.0%		
T2	7171 (17.6)	88.8%		2970 (17.0)	88.8%		
T3	22826 (56.0)	77.2%		9919 (56.8)	77.5%		
T4a	2579 (6.3)	61.6%		1038 (5.9)	60.7%		
T4b	1893 (4.6)	51.5%		796 (4.6)	49.6%		
**AJCC 8^th^ pN stage**			<0.001			<0.001	0.009
N0	23889 (58.6)	86.9%		10380 (59.5)	86.1%		
N1a	4978 (12.2)	78.9%		2182 (12.5)	79.1%		
N1b	5499 (13.5)	73.4%		2248 (12.9)	72.8%		
N2a	3416 (8.4)	63.5%		1495 (8.6)	65.6%		
N2b	2954 (7.3)	49.1%		1151 (6.6)	49.0%		
**AJCC 8^th^ TNM stage**			<0.001			<0.001	0.063
I	10810 (26.5)	91.7%		4585 (26.3)	90.8%		
IIA	11305 (27.8)	85.0%		5046 (28.9)	84.9%		
IIB	925 (2.3)	73.0%		365 (2.1)	70.8%		
IIC	849 (2.1)	66.5%		384 (2.2)	60.1%		
IIIA	2223 (5.5)	86.7%		972 (5.6)	87.2%		
IIIB	10632 (26.1)	72.9%		4526 (25.9)	73.0%		
IIIC	3992 (9.8)	47.9%		1578 (9.0)	47.8%		
**No. of rLNs**						<0.001	
< 12	10548 (25.9)	77.5%		4516 (25.9)	76.4%		
≥ 12	30188 (74.1)	80.1%		12940 (74.1)	80.3%		

AJCC, No., number; OS, overall survival; pT stage, pathological T stage; pN stage, pathological N stage; rLNs, retrieved lymph nodes. *P values of log-rank tests.

**Table 2:** Three- and five-year OS and 95% CI for AJCC 8th TNM classification and nTNM classification in training and validation cohorts

Variable	No. of patients (%)	HR (95% CI)	3-year OS	5-year OS	*P* value
***Training cohort***					
**TNM stage**					<0.001
I	10692 (26.2)	1 (Reference)	95.1%	91.7%	
IIA	11451 (28.1)	1.67 (1.57-1.77)	90.7%	85.0%	
IIB	911 (2.2)	2.82 (2.50-3.17)	82.0%	73.0%	
IIC	858 (2.1)	3.54 (3.16-3.97)	75.6%	66.5%	
IIIA	2202 (5.4)	1.36 (1.23-1.52)	92.1%	86.7%	
IIIB	10679 (26.2)	2.84 (2.68-3.01)	83.0%	72.9%	
IIIC	3943 (9.7)	6.33 (5.94-6.74)	60.8%	47.9%	
**nTNM staging system**					<0.001
I	9449 (23.2)	1 (Reference)	96.0%	93.2%	
IIA	11647 (28.6)	1.44 (1.31-1.59)	94.0%	89.9%	
IIB	6461 (15.9)	2.15 (1.98-2.34)	90.1%	83.7%	
IIC	5729 (14.1)	3.33 (3.05-3.63)	83.4%	73.7%	
IIIA	5410 (13.3)	4.61 (4.22-5.04)	76.7%	64.8%	
IIIB	920 (2.3)	6.86 (6.26-7.5)	65.1%	52.0%	
IIIC	1120 (2.7)	11.4 (10.2-12.7)	46.4%	34.7%	
***Validation cohort***					
**TNM classification**					<0.001
I	10692 (26.2)	1 (Reference)	95.0%	90.8%	
IIA	11451 (28.1)	1.60 (1.46-1.76)	91.4%	84.9%	
IIB	911 (2.2)	3.14 (2.62-3.77)	78.2%	70.8%	
IIC	858 (2.1)	4.22 (3.58-4.97)	70.9%	60.1%	
IIIA	2202 (5.4)	1.43 (1.23-1.68)	93.0%	87.2%	
IIIB	10679 (26.2)	2.77 (2.53-3.02)	83.2%	73.0%	
IIIC	3943 (9.7)	5.99 (5.43-6.60)	61.8%	47.8%	
**nTNM classification**					<0.001
I	9449 (23.2)	1 (Reference)	95.5%	91.6%	
IIA	11647 (28.6)	1.34 (1.16-1.54)	94.2%	89.8%	
IIB	6461 (15.9)	1.96 (1.74-2.22)	90.6%	83.7%	
IIC	5729 (14.1)	3.38 (2.96-3.85)	82.4%	72.4%	
IIIA	5410 (13.3)	4.26 (3.74-4.86)	76.7%	64.4%	
IIIB	920 (2.3)	6.14 (5.35-7.04)	66.2%	51.9%	
IIIC	1120 (2.7)	9.98 (8.45-11.8)	48.2%	35.8%	

CI, confidence interval; HR, hazard ratio; OS, overall survival; No., number; nTNM, novel TNM; TNM, tumor-node-metastasis.

**Table 3:** Prognostic performances of AJCC 8th TNM classification and nTNM classification in training and validation cohorts

Variables	AUC (95% CI)	AIC	P value^*^
**Training cohort**			<0.001
TNM classification	0.667 (0.662-0.672)	237741	
nTNM classification	0.678 (0.673-0.682)	236525	
**Validation cohort**			<0.001
TNM classification	0.660 (0.653-0.667)	92884	
nTNM classification	0.671 (0.664-0.678)	92414	

AUC, the areas under the curve; AIC, Akaike's information criterion; CI, confidence interval; nTNM, novel TNM; TNM, tumor-node-metastasis. *Hanley & McNeil tests of AUCs.

## Abbreviations

AJCC: American Joint Committee on Cancer
AIC: Akaike's information criterion
AUC: the areas under the curve
CI: confidence interval
CRC: colorectal cancer
DCA: decision curve analysis
HCC: hepatocellular cancer
HR: hazard ratio
IQR: interquartile range
OS: overall survival
SEER: Surveillance, Epidemiology, and End Results
TNM: tumor-node-metastasis
