# Supplementary material for: A Novel TNM Classification for Colorectal Cancers based on the Metro-ticket Paradigm
Source: J Cancer. 2021 Apr 5;12(11):3299–306. doi: 10.7150/jca.55097 (PMC8100802; doi:10.7150/jca.55097)
Supplement: Supplementary file 1 — Supplementary figures and tables. [file jcav12p3299s1.pdf]

Table S1. Five-year OS and 95% CI for AJCC 8th TNM classification and nTNM classification based on whether the retrieved lymph nodes were sufficient in training and validation cohorts.

**Variable**	**No. of retrieved lymph nodes < 12**			**No. of retrieved lymph nodes ≥ 12**		
	**HR (95% CI)**	**5-year OS**	***P* value**	**HR (95% CI)**	**5-year OS**	***P* value**
Training cohort						
TNM classification			<0.001			<0.001
I	1 (Reference)	90.3%		1 (Reference)	92.6%	
IIA	1.95 (1.77–2.15)	78.8%		1.68 (1.55–1.82)	86.8%	
IIB	2.98 (2.43–3.66)	66.4%		2.92 (2.52–3.39)	75.2%	
IIC	3.82 (3.12–4.69)	57.8%		3.69 (3.21–4.25)	69.2%	
IIIA	1.34 (1.14–1.58)	84.0%		1.41 (1.23–1.62)	88.1%	
IIIB	3.17 (2.89–3.48)	64.0%		2.92 (2.71–3.15)	75.5%	
IIIC	6.70 (5.89–7.62)	39.0%		7.00 (6.47–7.57)	49.1%	
nTNM classification			<0.001			<0.001
I	1 (Reference)	92.4%		1 (Reference)	93.8%	
IIA	1.61 (1.40–1.85)	87.2%		1.42 (1.24–1.62)	91.1%	
IIB	2.63 (2.33–2.96)	76.7%		2.14 (1.91–2.40)	85.9%	
IIC	3.73 (3.27–4.27)	66.5%		3.45 (3.05–3.89)	75.9%	
IIIA	5.45 (4.77–6.27)	53.7%		4.78 (4.23–5.39)	67.7%	
IIIB	7.97 (6.71–9.46)	40.0%		7.54 (6.68–8.50)	53.5%	
IIIC	10.3 (8.10–13.1)	35.2%		12.9 (11.3–14.8)	34.6%	
Validation cohort						
TNM classification			<0.001			<0.001
I	1 (Reference)	89.0%		1 (Reference)	91.8%	
IIA	2.04 (1.76–2.37)	78.1%		1.52 (1.35–1.72)	86.9%	
IIB	3.38 (2.50–4.57)	65.0%		3.14 (2.50–3.94)	73.1%	
IIC	6.24 (4.79–8.14)	41.7%		3.71 (3.01–4.58)	66.5%	
IIIA	1.63 (1.29–2.07)	83.7%		1.35 (1.10–1.65)	88.7%	
IIIB	3.35 (2.90–3.87)	63.4%		2.71 (2.42–3.04)	75.7%	
IIIC	6.93 (5.67–8.46)	37.6%		6.32 (5.60–7.13)	49.2%	
nTNM classification			<0.001			<0.001
I	1 (Reference)	90.8%		1 (Reference)	92.2%	
IIA	1.50 (1.21–1.85)	86.3%		1.30 (1.07–1.58)	91.3%	
IIB	2.45 (2.05–2.94)	77.2%		1.91 (1.61–2.25)	85.7%	
IIC	4.34 (3.56–5.29)	61.5%		3.21 (2.69–3.84)	76.1%	
IIIA	5.59 (4.55–6.87)	51.4%		4.18 (3.51–4.99)	67.8%	
IIIB	6.78 (5.17–8.90)	45.1%		6.62 (5.54–7.91)	52.8%	
IIIC	11.5 (8.21–16.2)	27.0%		10.6 (8.59–13.0)	37.4%	

CI, confidence interval; HR, hazard ratio; OS, overall survival; No., number; nTNM, novel TNM; TNM, tumor-node-metastasis.

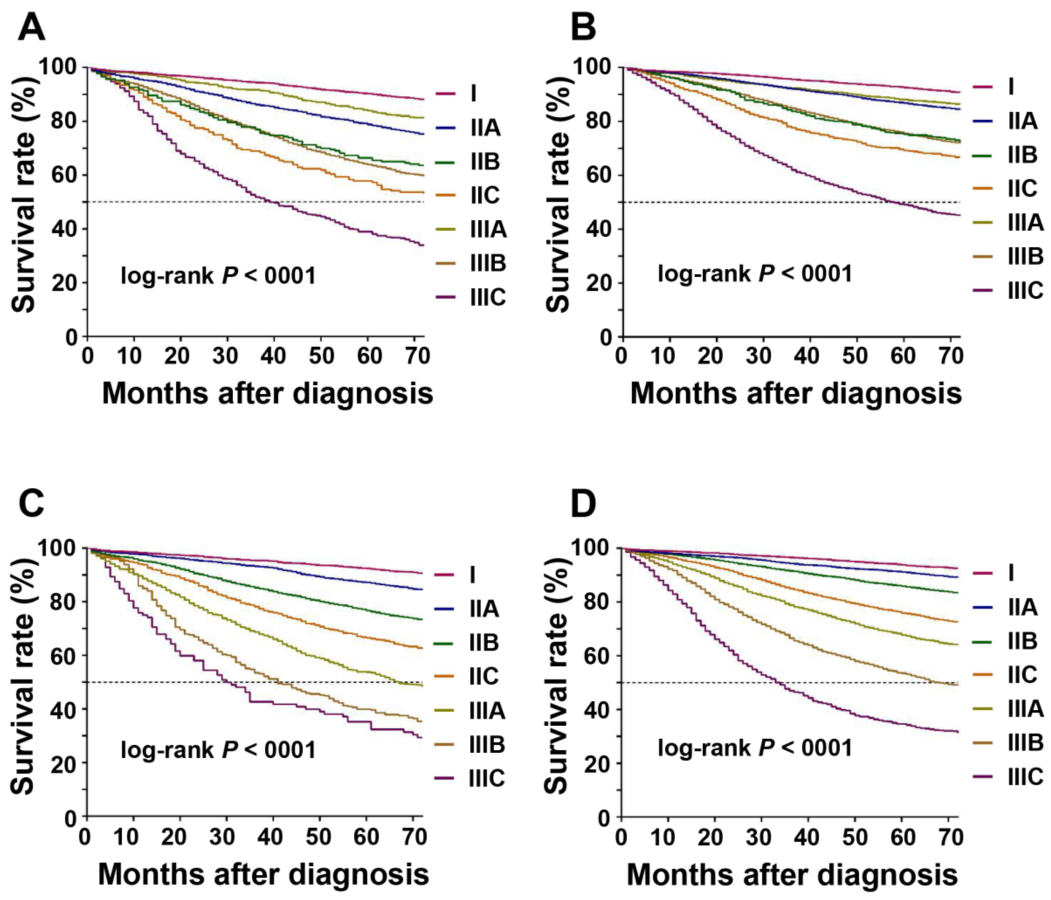

Figure S1. The Kaplan–Meier survival curve of the AJCC TNM and novel TNM (nTNM) staging systems based on whether the retrieved lymph nodes were sufficient in the training cohort. A: the AJCC TNM staging system in insufficient retrieved lymph nodes; B: the AJCC TNM staging system in sufficient retrieved lymph nodes; C: the nTNM staging system in insufficient retrieved lymph nodes; D: the nTNM staging system in sufficient retrieved lymph nodes.

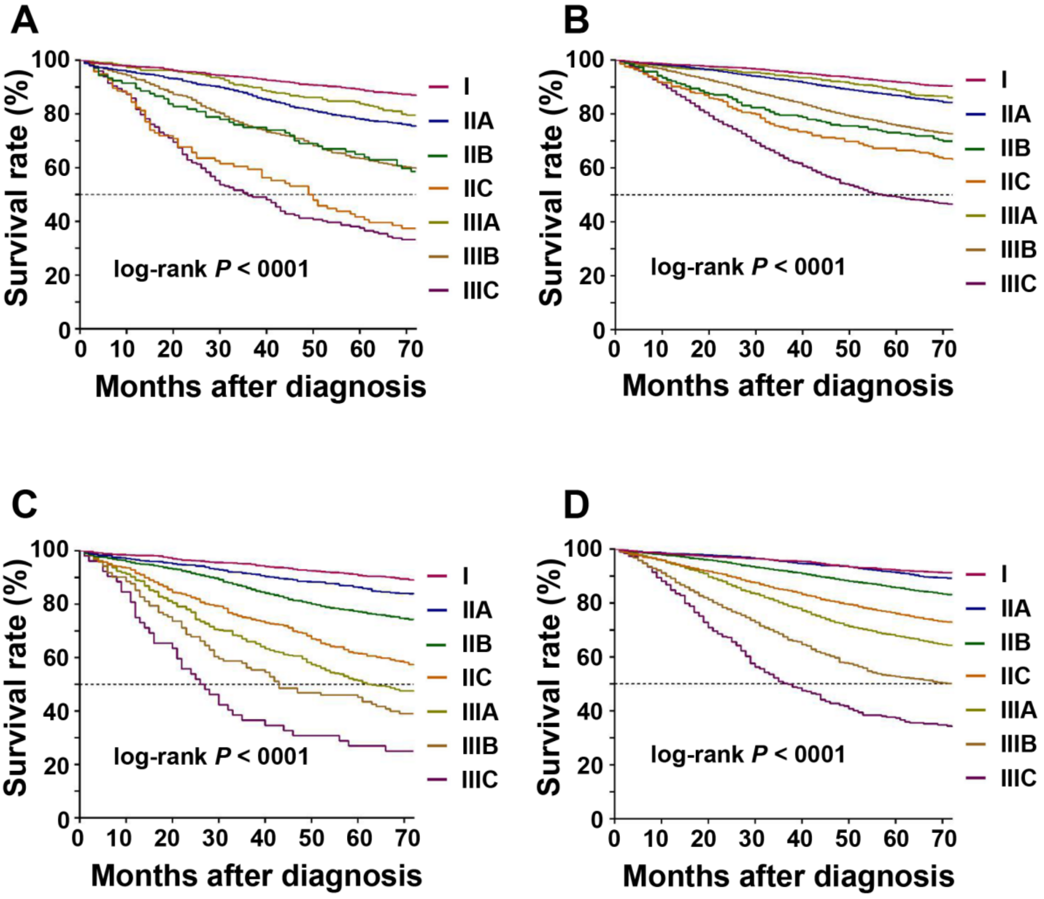

Figure S2. The Kaplan–Meier survival curve of the AJCC TNM and novel TNM (nTNM) staging systems based on whether the retrieved lymph nodes were sufficient in the validation cohort. A: the AJCC TNM staging system in insufficient retrieved lymph nodes; B: the AJCC TNM staging system in sufficient retrieved lymph nodes; C: the nTNM staging system in insufficient retrieved lymph nodes; D: the nTNM staging system in sufficient retrieved lymph nodes.
